# Distinctiveness of Reactive and Proactive Aggression from a Variable- and Person-based Approach in Juvenile Offenders and Community Youth

**DOI:** 10.1007/s10578-022-01479-5

**Published:** 2022-12-12

**Authors:** Lorena Maneiro, Aarón Argudo, Xosé Antón Gómez-Fraguela

**Affiliations:** Department of Clinical Psychology and Psychobiology, Universidade de Santiago de Compostela, C/ Xosé María Suárez Núñez, s/n, Campus Vida, 15782 Santiago de Compostela, Spain

**Keywords:** Reactive aggression, Proactive aggression, Behavioral profiles, Juvenile offenders, Adolescents

## Abstract

The goal of this study was to examine the distinctiveness of reactive aggression (RA) and proactive aggression (PA) from a variable- and person-based approach, their psychosocial correlates and behavioral outcomes, and analyze their replicability across two samples of adolescents. The forensic sample was composed of 231 juvenile offenders and the community sample included 321 youth. At a variable-based level, the results of the factor analysis supported the original two-factor model of aggression, and RA and PA showed differential associations with a set of psychosocial correlates and behavioral outcomes. At a person-based level, three subgroups were identified, namely low aggression, moderate RA, and mixed aggression. The mixed aggression group showed the most severe profile in both samples. These results support the distinctiveness of RA and PA at a variable-based level but lead to consider PA as a severity marker rather than a distinct subgroup at a person-based level.

## Introduction

The distinction between reactive aggression (RA) and proactive aggression (PA) has proven to be useful for understanding the underlying motivations of aggression in children and adolescents [1, 2]. Specifically, it has been proposed that RA emerges as a reaction of a perceived threat or provocation, guided by impulsive and emotional traits, whereas PA is an instrumental, “cold-blooded” behavior, intended to harm others [3, 4]. Despite different correlates, precursors and outcomes were found in relation to RA and PA from a variable-based approach [5, 6], it remains unclear whether this distinction can be useful for scientific and clinical practice [2, 7]. Some researchers argue that unique correlates or precursors do not necessarily reflects the existence of different groups of aggressive individuals [8, 9]. Thus, the identification of different subtypes of aggressive children and adolescents from a person-based approach becomes relevant to inform effective interventions tailored to their specific needs [1, 10].

### RA and PA from a Variable-Based Approach

The distinctiveness of RA and PA has been supported by several variable-based studies, mainly through factor analysis and differential correlates [6, 11, 12]. RA has been found to be strongly associated with temperamental and personality underpinnings such as impulsivity, anger and hostility, internalizing problems, as well as deficits in emotional regulation. On the other hand, PA has shown stronger relations with externalizing problems, delinquency, and individual characteristics such as psychopathic traits or positive outcome expectations [13, 14]. The variable-based approach assumes that these relationships are equal in all members of a group but does not take into account how aggressive traits are combined at the person level [10]. It is important to note that differential correlates of RA and PA may co-exist within individuals, contributing to a high overlap between both functions of aggression [9, 15]. Indeed, the high correlation that was found between RA and PA led to a huge criticism about their actual distinction and if it is possible to identify different profiles of aggressive individuals [15, 16]. Using analytic techniques that allow controlling for the co-occurrence of both functions of aggression may contribute to support the usefulness of their distinction in clinical practice [17, 18].

### RA and PA from a Person-Based Approach

RA and PA tend to co-occur within individuals but it is unlikely that all individuals show the same behavioral pattern [18]. The person-based approach proposes the existence of different profiles of individuals who may be grouped based on particular characteristics [15]. Accordingly, prior studies have tried to answer the question of whether it is possible to distinguish different subgroups of individuals with predominantly RA or PA, or if they only differ in severity [8, 17]. Overall, findings support the existence of a low aggression group, a predominantly RA group, and a mixed group, but not a “purely” proactive profile. These results were replicated in clinical and community samples of children [9, 16, 19]; detained adolescents and community youth [17, 18, 20, 21], and adults [15]. Thus, some authors argued that PA can be seen as a severity marker rather than an indicator of a distinct subgroup [8, 9, 20].

Nevertheless, other solutions were found in adolescent samples that were slightly different. For instance, Smeets et al. [8] conducted an exploratory factor analysis (EFA) of the Reactive and Proactive Questionnaire (RPQ) [6] to examine alternative factor solutions of the scale. Using a clinical sample of adolescents, these authors found an alternative three-factor solution corresponding to PA, RA internal frustration, and RA external provocation. Despite the results of the latent profile analysis (LPA) showed a better fit for a four-class model, this solution followed the same trend as the previously described profiles, with one more group defined by moderate levels in both RA and PA. On the other hand, four groups were identified in a sample of middle school students, namely RA, PA, proactive/reactive aggressive, and uninvolved, with a clearly defined PA group [22]. In the same line, van Dijk et al. [9] supported the existence of both RA and PA subtypes, however, they rejected the “pure” model in favor of a “both subtypes” model, in which individuals in reactive and proactive groups also displayed the other function of aggression, although to a lesser extent.

### Psychosocial and Behavioral Profiles from a Person-Based Approach

Most of the studies conducted with adolescent samples did not support the existence of differential behavioral profiles for the indicated classes but they supported a severity model. Specifically, no significant differential associations between aggressive profiles and external correlates emerged as regards internalizing and externalizing problems, empathy, or social competence [8, 9, 17, 21]. On the contrary, the mixed group, which scored higher in both RA and PA, showed the most severe risk profile, including more ADHD, oppositional defiant and conduct disorders, higher levels of anger dysregulation, impulsivity, callous-unemotional traits, and lower empathy [8, 17, 18, 21, 22]. Despite no significant differences were found between the mixed group and the RA group in specific psychosocial correlates and behavioral outcomes, the former usually obtain higher scores, indicating that both groups seems to differ in terms of severity of risk factors rather than in the type of risk factor [21].

### The Current Study

Previous studies have tried to examine whether RA and PA are meaningful distinctions from a variable- and person-based approach. Despite empirical support for the distinction between both functions of aggression at a variable-based level, some studies at a person-based level did not support the existence of a “proactive-only” group. A few studies were conducted with adolescent samples [8, 17, 18, 20, 21], which mostly differ in the type of sample, measure of aggression, and analytic technique. Therefore, more studies are needed to further investigate the distinctiveness of RA and PA and delve into risk profiles, correlates, and outcomes. The identification of aggressive profiles will assist in identifying those individuals in need of intensive interventions as well as in the adaptation of treatments to specific profiles. Thus, the goal of the current study is to analyze the distinctiveness of RA and PA from a variable- and a person-based approach and examine the associations with a set of psychosocial correlates (i.e., antisocial peers, attitudes towards violence, impulsivity, psychopathic traits) and behavioral outcomes (i.e., rule-breaking behavior, theft, vandalism, drug problems) in two samples of adolescents. In addition, this study aims at analyzing differences between juvenile offenders and community youth and determine whether the aggressive profiles may be replicated in both samples. The majority of studies in the field have used analytic strategies such as cluster analysis, median splits or cut-off scores to identify groups of aggressive individuals, considering only one sample from a specific population. The current study overcomes these limitations by using mixture models for profiles identification while considering two different samples of adolescents involved in the juvenile justice system and in community settings.

The following hypotheses are proposed. From a variable-based approach, two factors of RA and PA that show differential associations with a set of psychosocial correlates and behavioral outcomes are expected. Specifically, RA is expected to show stronger associations with impulsivity traits and the reactive facet of attitudes towards violence. On the other hand, PA is expected to show stronger associations with antisocial peers, psychopathic traits, antisocial behavior, and the attitudes towards violence facet related to culture of violence. From a person-based approach, three distinct profiles are expected to be identified in line with previous studies, namely a low aggression group, a predominantly RA group, and a mixed group high in RA and PA. We expect to find support for the severity model, in which the mixed aggression group would show the highest risk in both psychosocial correlates and behavioral outcomes. Finally, and given these profiles were previously found in both forensic and community samples of adolescents, these results are expected to be replicated in the sample of juvenile offenders and community youth.

## Methods

### Participants

Data used in this study are part of a broader research project (i.e., juvenile offender’s risk assessment), focused on the analysis of risk and protective factors of antisocial behavior in adolescents [23]. For the purposes of the current study, two independent samples of forensic and community youth were selected. Participants were included only if they were 14 years old or close to this age (i.e., less than six months to 14). Males, females, and non-binary youth were considered for their inclusion in this study. Regarding the forensic sample, youth who had been evaluated by means of the VRAI protocol [23] during the initial assessment were considered for participation. Adolescents with intellectual disabilities that could hamper their understanding of the questionnaire were excluded. The forensic sample was initially composed of 237 juvenile offenders (73.8% males) aged 14–21 (*M* = 16.77, *SD* = 2.22), from three juvenile justice agencies in Spain. Six cases were removed from the analysis because they had missing data in all the variables of study, giving rise to a final sample of 231 juvenile offenders (73.2% males), aged 14–21 (*M* = 16.75, *SD* = 2.38). Of these, 52.8% of participants were born in Spain, 6.9% were from South America, 3.9% were Africans, and 2.6% came from other European countries. The remaining 33.8% did not provide information on this variable.

The initial community sample was composed of 324 adolescents (41.4% males) aged 13–21 (*M* = 16.27, *SD* = 1.77). After removing three cases with missing data in all the variables of study, the final sample was composed of 321 adolescents (41.7% males), aged 13–21 (*M* = 16.26, *SD* = 1.77). Of these, 61.3% were born in Spain, 1.6% came from South America, 1.3% came from other European countries, and 35.8% did not provide information on this variable.

### Variables and Measures

**Aggression**. The functions of aggression were assessed by means of the Spanish version of the Reactive and Proactive Questionnaire (RPQ) [6, 24]. The self-reported RPQ is composed of 23 items, scored on a 3-point scale from 0 (*never*) to 2 (*often*), intended to assess both RA (11 items, e.g. “I reacted angrily when provoked by others”) and PA (12 items, e.g., “I had fights with others to show who was on top”).

**Antisocial peers**. The Deviant Peer Scale (DPS) [25] was used to measure the presence of antisocial behavior in the adolescent’s peer group. The self-reported DPS is composed of 12 items, rated on a 4-point scale from 0 (*never*) to 3 (*often*). The global scale assesses two facets of antisocial behavior in the peer group: (1) an antisocial factor composed of 8 items related to general antisocial behavior (e.g., “my friends get into trouble in their free time”) and (2) a drug factor composed of 4 items involving deviant behavior specifically related to drug use (e.g., “my friends know how to get drugs”). For the purposes of the current study, only the antisocial factor was considered in the analyses.

**Attitudes towards violence**. The Attitudes Toward Violence Scale (ATV) [26] was used to measure antisocial attitudes in adolescents. The self-reported ATV is composed of 14 items which are grouped in two factors: (1) a factor of culture of violence, which reflects the identification with violence as a valued activity (7 items, e.g., “it’s ok to use violence to get what you want”), and (2) a factor of reactive violence, which refers to the justification of the use of violence as a response to actual or perceived threats (7 items, e.g., “if a person hits you, you should hit them back”). The items were rated on a four-point scale from 0 (*never*) to 3 (*often*).

**Impulsivity traits**. Impulsivity was assessed by means of the short Spanish version of the UPPS-P [27]. This scale is composed of 20 items referring to 5 impulsivity facets: positive urgency (4 items, e.g., “I tend to act without thinking when I am really excited”), negative urgency (4 items, e.g., “when I am upset I often act without thinking”), (lack of) premeditation (4 items, e.g., “I usually think carefully before doing anything”), (lack of) perseverance (4 items, e.g., “I finish what I start”), and sensation seeking (4 items, e.g., “I quite enjoy taking risks”). The items were scored on a four-point scale ranging from 1 (*totally disagree*) to 4 (*totally agree*).

**Psychopathic traits**. The Spanish version of the Youth Psychopathic Traits Inventory-Short Version (YPI-S) [28] was used to evaluate the three facets of psychopathy, namely, grandiose–manipulative (GM, e.g., “it’s easy for me to manipulate people”), callous–unemotional (CU, e.g., “to be nervous and worried is a sign of weakness”), and impulsive–irresponsible (IMP, e.g., “I consider myself as a pretty impulsive person”). The YPI-S is composed of 18 items (6 items each facet) scored on a 4-point scale ranging from 0 (*does not apply at all*) to 3 (*apply very well*).

**Antisocial behavior**. Four scales of the Antisocial Behavior Questionnaire (ABQ) [29] were used to measure the frequency of several types of problematic behavior, including rule-breaking behavior (e.g., “spending the night out without permission”), theft (e.g., “taking something from class without permission with the intention of stealing it”), vandalism (e.g., “setting fire to something: a dustbin, table, car, etc.”), and drug problems (e.g., “being arrested for drug possession). Each scale is composed of 6 items scored on a four-point scale from 0 (*never*) to 3 (*often*).

### Procedure

This research was conducted in accordance with the ethical standards of the Bioethics Committee at the University of Santiago de Compostela (Spain), following the General Data Protection Regulation (GDPR) and the tenets of the Declaration of Helsinki. This study is part of a broader ongoing research project that is being carried out in collaboration with a set of juvenile justice centers located in three different regions in Spain (i.e., Galicia, Principado de Asturias, and Balearic Islands). The main goal of the project is to analyze the risk and protective factors of antisocial behavior and delve into specific needs of adolescents from different settings (e.g., forensic, community). To that end, an online survey was designed to collect data by including a set of previously well-validated questionnaires intended to assess different factors from distinct domains (e.g., family, individual, school, peers). Regarding the forensic sample, youth completed the survey as part of a clinical protocol that is conducted during the initial assessment after the admission to the youth center. Technical staff were responsible for presenting the project to the juvenile offenders and monitoring them while filling out the questionnaire. The community sample was recruited through personal contacts of students as part of Psychology and Criminology courses. According to the Spanish data protection regulations, adolescents aged 14 or older are able to provide their own informed consent for participation, thus, age was considered as an inclusion criterion for participation in the study. In addition, informed assent of all adolescents, both from the juvenile justice system and from the community, was requested before the beginning of the survey. Confidentiality and anonymity were ensured throughout the research project following the ethical guidelines.

### Data Analysis

Descriptive statistics and differences between juvenile offenders and community youth were analyzed. Cohen’s *d* was the effect size estimator used for the analysis of the magnitude of differences between groups. An effect size of 0.2 was considered small, 0.5 was considered medium, and 0.8 was considered large [30]. A Confirmatory Factor Analysis (CFA) was conducted in Mplus 7.4 [31], with robust weighted least squares used as estimator (WLSMV). The original two-interrelated factor model was specified including the 23 items as observed variables and two correlated latent factors of RA and PA. Given a three-factor solution of the 23-items of the RPQ has been previously found [8], this model was also tested for comparative reasons. Model fit was assessed using root-mean-square error of approximation (RMSEA), comparative fit index (CFI), and the Tucker-Lewis index (TLI). RMSEA values lower or equal to 0.06, and TLI and CFI values of 0.95 or higher were considered indicators of good model fit [32]. A multigroup factor analysis was conducted to test for configural, metric, and scalar invariance, to examine whether the factor structure, factor loadings, and item intercepts held across groups (i.e., juvenile offenders, community youth). To support the distinctiveness of RA and PA from a variable-based approach, zero-order and partial correlations controlling for the other function of aggression were analyzed regarding psychosocial correlates and behavioral outcomes.

A series of latent profile analyses (LPA) were conducted in Mplus 7.4, including RA and PA as latent profile indicators. Independent LPAs were analyzed for the forensic and community samples. The best solution was selected according to empirical criteria. Lower Bayesian Information Criteria (BIC) and entropy values closer to 1 were indicative of better fit. In addition, significant Bootstrap Likelihood Ratio Test (BLRT) indicated whether a *k* class model significantly improved the model with one class less, therefore, solutions obtaining significant BLRT values were preferred. Differences between subgroups as regards class indicators (i.e., RA and PA) were analyzed through a set of ANOVAs in SPSS 25, using the Tukey–Kramer index for post hoc comparisons. Partial eta squared was used as the effect size estimator, considering 0.01 to 0.06 small, 0.06 to 0.14 medium and 0.14 or higher large [30]. Finally, differences among subgroups in psychosocial correlates and behavioral outcomes were analyzed through the improved BCH method in Mplus 7.4, a 3-step procedure for continuous covariates as distal outcomes.

## Results

### Descriptive Statistics and Differences Between Forensic and Community Samples

Descriptive statistics and differences between juvenile offenders and community youth are displayed in Table 1. The results of the *t*-test comparisons for independent samples showed significant differences between juvenile offenders and community youth in all the study variables. Juvenile offenders scored higher in all the variables, although the magnitude of the differences varied among them. Specifically, the largest differences were found in rule-breaking behavior and drug problems, followed by theft. Moderate differences were found between groups in aggression (i.e., RA and PA), antisocial peers, attitudes towards violence (i.e., culture of violence and reactive violence), psychopathic traits (i.e., CU and IMP), and vandalism. The smallest differences emerged regarding impulsivity facets and, more specifically, in positive and negative urgency, (lack of) perseverance, and sensation seeking.

**Table 1:** Descriptive Statistics and Differences Between Juvenile Offenders and Adolescents from the General Population in all the Study Variables

	Total sample		Juvenile offenders		Community youth		*t*	Cohen’s *d*
	Cronbach’s α	*M* (*SD*)	Cronbach’s α	*M* (*SD*)	Cronbach’s α	*M* (*SD*)		
Reactive aggression	0.86	10.68 (6.26)	0.85	12.54 (6.46)	0.86	9.34 (5.76)	5.98***	0.52
Proactive aggression	0.92	3.72 (5.71)	0.89	5.42 (6.18)	0.93	2.41 (4.96)	5.39***	0.55
Antisocial peers	0.91	4.14 (4.62)	0.93	5.25 (5.56)	0.87	3.33 (3.61)	4.59***	0.41
Attitudes towards violence								
Culture of violence	0.77	3.81 (3.67)	0.78	5.13 (4.10)	0.73	2.85 (2.99)	7.19***	0.64
Reactive violence	0.83	7.57 (4.74)	0.81	9.19 (4.97)	0.81	6.40 (4.19)	6.93***	0.61
Impulsivity traits								
Positive urgency	0.73	4.40 (2.66)	0.73	4.76 (2.81)	0.72	4.13 (2.51)	2.74**	0.24
Negative urgency	0.83	4.98 (3.09)	0.80	5.28 (3.12)	0.85	4.76 (3.05)	1.97*	0.17
(lack of) premeditation	0.81	5.95 (2.74)	0.76	6.49 (2.66)	0.83	5.56 (2.72)	4.01***	0.35
(lack of) perseverance	0.80	5.15 (2.65)	0.77	5.60 (2.59)	0.83	4.84 (2.66)	3.32***	0.29
Sensation seeking	0.79	5.01 (2.95)	0.77	5.33 (3.05)	0.82	4.78 (2.86)	2.16*	0.19
Psychopathic traits								
GM	0.82	4.77 (4.12)	0.85	5.49 (4.59)	0.79	4.26 (3.66)	3.36***	0.30
CU	0.64	4.28 (3.05)	0.57	5.08 (3.06)	0.67	3.70 (2.90)	5.39***	0.46
IMP	0.73	7.26 (3.70)	0.70	8.25 (3.78)	0.75	6.55 (3.48)	5.39***	0.47
Behavioral outcomes								
Rule-breaking behavior	0.87	3.54 (4.51)	0.86	5.91 (5.14)	0.82	1.86 (3.05)	10.62***	0.96
Theft	0.88	1.95 (3.63)	0.88	3.56 (4.64)	0.81	0.81 (2.01)	8.43***	0.77
Vandalism	0.82	1.83 (3.04)	0.82	2.84 (3.75)	0.77	1.11 (2.16)	6.27***	0.57
Drug problems	0.89	3.54 (5.20)	0.90	6.02 (6.38)	0.80	1.78 (3.15)	9.23***	0.84

Note. GM = Grandiose/manipulative; CU = Callous/unemotional, IMP = Impulsive/irresponsible.

* p < .05, ** p < .01, *** p < .001

### Factor Analysis and Measurement Invariance

The results of the CFA for the original two-interrelated factor model showed a good model fit (RMSEA = 0.06 [0.06, 0.07], CFI = 0.96, TLI = 0.95). With regards to the three-factor structured proposed by Smeets et al. (2017), which split the RA in two factors (i.e., RA internal frustration and RA external provocation), the results also showed a good model fit (RMSEA = 0.06 [0.06, 0.07], CFI = 0.96, TLI = 0.95). Given the fit indices of both two-factor and three-factor models were almost equal, the original factor structure of the RPQ was selected for subsequent analyses. A CFA of the two-factor model was then analyzed separately for the forensic and community samples. The results of the CFA indicated a good model fit for the forensic (RMSEA = 0.05 [0.04, 0.06], CFI = 0.98, TLI = 0.97) and community samples (RMSEA = 0.05 [0.05, 0.06], CFI = 0.98, TLI = 0.98). To test for measurement invariance across groups (i.e., juvenile offenders and community youth), configural, metric and scalar invariance were examined in sequence. Model fit indices for the configural model were RMSEA = 0.06 [0.05, 0.06], CFI = 0.98, and TLI = 0.98; for the metric model were RMSEA = 0.06 [0.06, 0.07], CFI = 0.97, and TLI = 0.97; and for the scalar model were RMSEA = 0.06 [0.06, 0.07], CFI = 0.97, and TLI = 0.97. The invariance testing results evidenced significant differences between groups in the configural (χ^2^_(458)_ = 880.83, *p* < .001), metric (χ^2^_(479)_ = 987.69, *p* < .001), and scalar models (χ^2^_(523)_ = 1059.79, *p* < .001), indicating that the factor structure, factor loadings, and item intercepts were not invariant across samples.

### Association Between RA and PA with Psychosocial Correlates and Behavioral Outcomes

The results of the zero-order and partial correlations between RA and PA with psychosocial correlates and behavioral outcomes are displayed in Table 2. Significant zero-order correlations were found between RA and PA with all the variables of study in both samples, except for the relationships with (lack of) premeditation and (lack of) perseverance in the sample of juvenile offenders. Partial correlations controlling for the other function of aggression showed, though, a different pattern of results. Regarding the forensic sample, after controlling for the effect of PA, RA remained significantly associated with attitudes towards violence, positive and negative urgency, sensation seeking, and grandiose/manipulative and impulsive psychopathic traits. However, the associations of RA with behavioral outcomes, antisocial peers, and callous/unemotional traits turned out non-significant. On the other hand, all the associations of PA remained significant after controlling for the effect of RA, except for negative urgency and the impulsivity facet of psychopathy. The results in the community sample were slightly different. RA remained significantly associated with all the variables but theft, vandalism, and drug problems, after controlling for PA. The relationships between PA and negative urgency, (lack of) premeditation, (lack of) perseverance, and the impulsivity facet of psychopathy, turned out non-significant when controlling for the effect of RA.

**Table 2:** Zero-order and Partial Correlations of Reactive and Proactive Aggression with Psychosocial Correlates and Behavioral Outcomes According to Sample Type

	Juvenile offenders		Community youth	
	RA	PA	RA	PA
Antisocial peers	0.49*** (0.09)	0.65*** (0.50***)	0.59*** (0.18**)	0.69*** (0.49***)
Attitudes towards violence				
Culture of violence	0.67*** (0.33***)	0.75*** (0.55***)	0.65*** (0.18**)	0.81*** (0.64***)
Reactive violence	0.63*** (0.39***)	0.60*** (0.30***)	0.64*** (0.38***)	0.59*** (0.26***)
Impulsivity traits				
Positive urgency	0.66*** (0.48***)	0.54*** (0.17*)	0.58*** (0.36***)	0.51*** (0.17**)
Negative urgency	0.68*** (0.60***)	0.42*** (-0.07)	0.67*** (0.58***)	0.43*** (-0.10)
(lack of) premeditation	0.09 (0.12)	0.01 (-0.07)	0.32*** (0.20**)	0.26*** (0.04)
(lack of) perseverance	0.03 (0.00)	0.04 (0.03)	0.27*** (0.15*)	0.25*** (0.07)
Sensation seeking	0.43*** (0.22**)	0.41*** (0.18*)	0.39*** (0.18**)	0.39*** (0.17**)
Psychopathic traits				
GM	0.58*** (0.25***)	0.65*** (0.43***)	0.54*** (0.23***)	0.57*** (0.31***)
CU	0.20** (-0.03)	0.32*** (0.26***)	0.50*** (0.19**)	0.54*** (0.30***)
IMP	0.70*** (0.57***)	0.49*** (0.03)	0.66*** (0.49***)	0.51*** (0.08)
Behavioral outcomes				
Rule-breaking behavior	0.51*** (0.15)	0.63*** (0.45***)	0.63*** (0.21***)	0.74*** (0.53***)
Theft	0.44*** (-0.01)	0.66*** (0.54***)	0.55*** (-0.07)	0.81*** (0.71***)
Vandalism	0.45*** (-0.06)	0.71*** (0.62***)	0.55*** (0.04)	0.75*** (0.60***)
Drug problems	0.39*** (0.09)	0.49*** (0.34***)	0.47*** (-0.03)	0.69*** (0.56***)

Note. Partial correlations controlling for the effect of the other function of aggression are displayed in parentheses. RA = reactive aggression; PA = proactive aggression; GM = grandiose/manipulative; CU = callous/unemotional, IMP = impulsive/irresponsible

* p < .05, ** p < .01, *** p < .001

### Identification of Subgroups Based on RA and PA

**Fig. 1:**
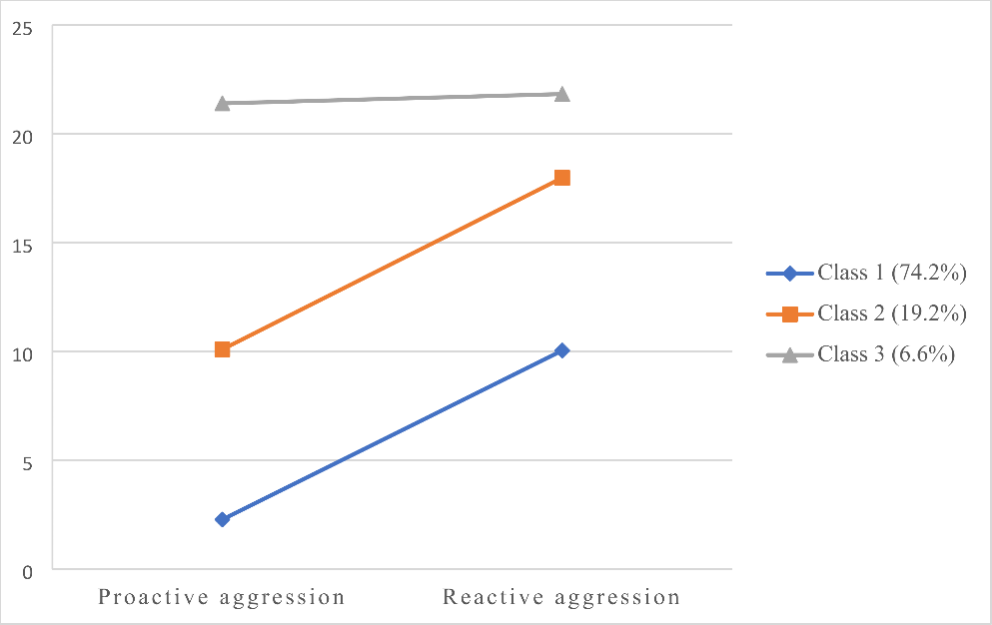
Mean Scores in Latent Profile Indicators for the Three-class Solution in the Sample of Juvenile Offenders

**Fig. 2:**
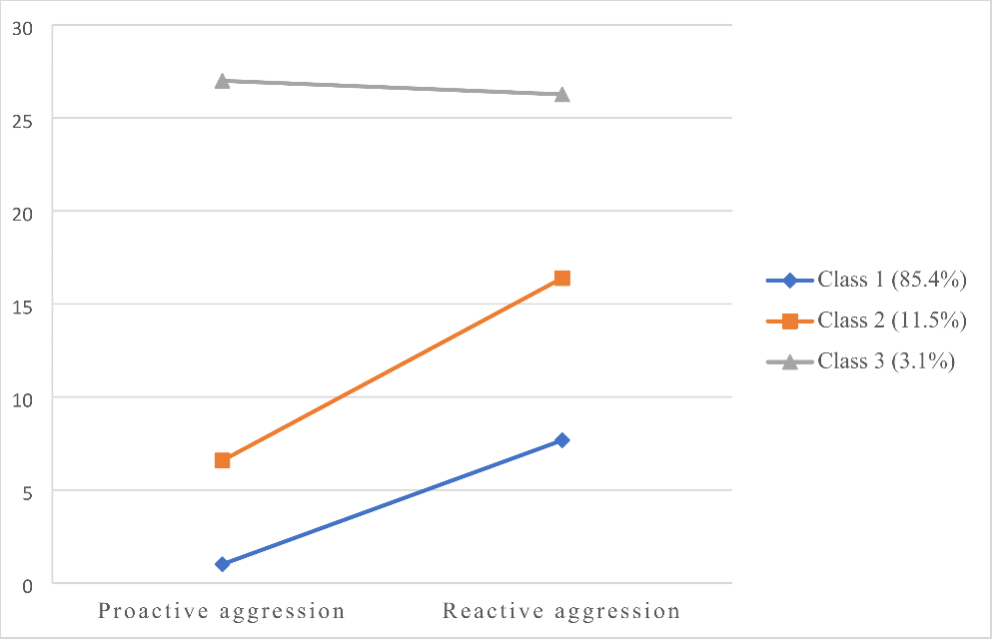
Mean Scores in Latent Profile Indicators for the Three-class Solution in the Sample of Adolescents from the General Population

The results of the LPA from one to four classes for both forensic and community samples are presented in Table 3. In the case of the forensic sample, the results favored the three-class model over the one- and two-class models. Furthermore, the three-class model obtained a lower BIC and higher entropy compared to the four-class model and was, thus, considered the best solution. The three-class solution identified three different profiles based on the levels of RA and PA: a low aggression group (74.2%), a moderate RA (19.2%), and a mixed aggression subgroup (6.6%). The three profiles are displayed in Fig. 1. Regarding the community sample, the results of the LPA followed a similar trend. The fit indices favored the three-class model over the one- and two-class solutions, however, the four-class model obtained a lower BIC than the three-class and still a high entropy and significant BLRT values. Nevertheless, one class in the four-class solution included less than 1% of the total sample and the classification probabilities for two classes were very low (0.59 and 0.76 for class 2 and class 3, respectively). Thus, the three-class model was considered the best solution. In this case, 85.4% of the sample of community youth was classified in the low aggression group, 11.5% in the moderate RA group, and 3.1% in the mixed aggression group. The three profiles identified in the community sample are presented in Fig. 2.

**Table 3:** Fit Indices for Latent Profile Models Including Reactive and Proactive Aggression as Indicators

Number of classes	BIC	Entropy	BLRT
Forensic sample			
One-class	2716.54	-	-
Two-class	2587.43	0.88	145.41***
**Three-class**	**2542.11**	**0.86**	61.62***
Four-class	2544.42	0.69	13.99*
Community sample			
One-class	3494.09	-	-
Two-class	3160.47	0.97	350.93***
**Three-class**	**3055.71**	**0.91**	122.08***
Four-class	3052.34	0.90	20.69***

Note. BIC = Bayesian Information Criteria; BLRT = Bootstrap Likelihood Ratio Test. Results in bold are considered the best model solutions

* p < .05, *** p < .001

Descriptive statistics and differences among subgroups in latent class indicators (i.e., RA and PA) are shown in Table 4 separated by sample. Regarding the forensic sample, significant differences were found among subgroups in both RA and PA, except between the moderate RA and mixed aggression subgroups, which did not differ in the levels of RA. The differences found were mainly quantitatively, that is, the low aggression subgroup obtained the lowest scores in both functions of aggression, the mixed aggression obtained the highest scores and the moderate RA scored in the middle. However, the magnitude of the differences among subgroups was larger for PA. The results in the community sample followed the same trend and evidenced quantitative and significant differences in both RA and PA among the three groups. Similarly, the low aggression group obtained the lowest scores, the moderate RA scored in the middle, and the mixed-aggression subgroup obtained the higher scores. The magnitude of the differences was also larger for PA.

**Table 4:** Descriptive Statistics and Differences Among Subgroups in Reactive and Proactive aggression

	Low aggression M (SD)	Moderate RA M (SD)	Mixed Aggression M (SD)	*F*	Partial eta squared
Forensic sample					
Reactive aggression	10.09 (4.86) _a_	18.81 (5.34) _b_	22.00 (4.00) _b_	85.05***	0.43
Proactive aggression	2.24 (1.94) _a_	10.24 (2.50) _b_	21.50 (3.62) _c_	610.26***	0.87
Community sample					
Reactive aggression	7.68 (3.85) _a_	17.05 (3.67) _b_	26.47 (4.57) _c_	198.13***	0.56
Proactive aggression	1.01 (1.25) _a_	6.67 (2.58) _b_	27.00 (4.96) _c_	847.37***	0.88

*** *p* < .001

Means with different subscripts (a, b, c) were significantly different (p < .05) in post hoc pairwise comparisons (subscript *a* represents the lowest score/s in the analyzed indicator)

### Differences Among Subgroups in Psychosocial Correlates and Behavioral Outcomes

Firstly, a three-step procedure where covariates predict the latent class was used to analyze whether the three profiles differed in gender composition. Using the low aggression group as the reference class, the results in the forensic sample did not show significant differences in the gender composition compared to the moderate RA (*B* = 0.19, *p* = .697) and the mixed aggression subgroups (*B* = 0.33, *p* = .640). There were no significant differences between the moderate RA and the mixed aggression groups (*B* = 0.13, *p* = .876). Nevertheless, in the community sample the low aggression group included more females than the moderate RA (*B* = 1.08, *p* = .014), but no significant differences were found between the low aggression and mixed aggression (*B* = 0.60, *p* = .478), and the moderate RA and mixed aggression (*B* = 1.68, *p* = .081). In terms of age, the results of the BCH method showed no significant differences among subgroups either in the forensic sample or in the community sample.

Comparisons among subgroups regarding psychosocial correlates and behavioral outcomes in the sample of juvenile offenders are shown in Table 5. Significant differences among subgroups were found in all the variables except for (lack of) premeditation and (lack of) perseverance. Post-hoc comparisons evidenced significant differences among all the three subgroups in antisocial peers, attitudes towards violence, grandiose/manipulative psychopathic traits, rule-breaking behavior, theft, and vandalism, showing the mixed aggression group the highest scores, the moderate RA the middle scores, and the low aggression group the lowest scores. However, no significant differences were found between the mixed aggression and moderate RA subgroup in positive and negative urgency, sensation seeking, callous/unemotional, the impulsivity facet of psychopathy, and drug problems.

**Table 5:** Comparisons Among Subgroups Regarding Psychosocial Correlates and Behavioral Outcomes in the Sample of Juvenile Offenders

	Low aggression (n = 170, 74.2%) *M* (*SE*)	Moderate RA (n = 44, 19.2%) *M* (*SE*)	Mixed aggression (n = 15, 6.6%) *M* (*SE*)	χ^2^
Age	16.62 (0.22) _a_	17.03 (0.28) _a_	17.09 (0.38) _a_	1.802
Antisocial peers	3.33 (0.31) _a_	8.36 (1.08) _b_	15.315 (1.70) _c_	67.51***
Attitudes towards violence				
Culture of violence	3.41 (0.22) _a_	8.17 (0.67) _b_	13.03 (0.97) _c_	134.22***
Reactive violence	7.50 (0.34) _a_	12.78 (0.79) _b_	15.68 (0.34) _c_	89.83***
Impulsivity traits				
Positive urgency	3.72 (0.18) _a_	6.95 (0.47) _b_	8.61 (0.71) _b_	80.43***
Negative urgency	4.39 (0.23) _a_	7.34 (0.54) _b_	8.27 (0.60) _b_	53.62***
(lack of) premeditation	6.40 (0.21) _a_	7.08 (0.48) _a_	5.77 (0.89) _a_	2.00
(lack of) perseverance	5.60 (0.21) _a_	5.30 (0.47) _a_	6.27 (0.71) _a_	1.19
Sensation seeking	4.56 (0.23) _a_	7.02 (0.53) _b_	7.87 (0.65) _b_	36.34***
Psychopathic traits				
GM	3.91 (0.32) _a_	8.49 (0.60) _b_	11.68 (1.25) _c_	76.17***
CU	4.54 (0.23) _a_	5.94 (0.54) _b_	7.41 (0.92) _b_	14.110***
IMP	7.10 (0.27) _a_	10.64 (0.58) _b_	12.45 (0.90) _b_	56.62***
Behavioral outcomes				
Rule-breaking behavior	4.06 (0.35) _a_	10.10 (0.82) _b_	13.14 (1.21) _c_	89.44***
Theft	1.84 (0.28) _a_	6.94 (0.87) _b_	10.49 (1.08) _c_	85.48***
Vandalism	1.42 (0.20) _a_	5.42 (0.75) _b_	10.07 (1.08) _c_	87.97***
Drug problems	4.05 (0.43) _a_	10.71 (1.27) _b_	12.94 (1.66) _b_	47.71***

Note. GM = Grandiose/manipulative; CU = Callous/unemotional, IMP = Impulsive/irresponsible. Means with different subscripts (a, b, c) were significantly different (p < .05) in post hoc pairwise comparisons (subscript *a* represents the lowest score/s in the analyzed indicator)

*** p < .001

Comparisons among subgroups regarding psychosocial correlates and behavioral outcomes in the sample of community youth are shown in Table 6. Significant differences among subgroups were found in all the variables of study. Post-hoc comparisons indicated that the three groups differed among them in all the variables but negative urgency, (lack of) premeditation, and (lack of) perseverance. Specifically, the mixed aggression group evidenced the highest scores, the moderate RA scored in the middle, and the low aggression obtained the lowest scores. No significant differences were found between the moderate RA and the mixed aggression group in negative urgency and (lack of) premeditation. However, only the low aggression and the moderate RA group differed in terms of (lack of) perseverance.

**Table 6:** Comparisons Among Subgroups Regarding Psychosocial Correlates and Behavioral Outcomes in the Sample of Community Youth

	Low aggression (n = 274, 85.4%) *M* (*SE*)	Moderate RA (n = 37, 11.5%) *M* (*SE*)	Mixed aggression (n = 10, 3.1%) *M* (*SE*)	χ^2^
Age	16.32 (0.12) _a_	15.77 (0.25) _a_	16.69 (0.50) _a_	4.36
Antisocial peers	2.42 (0.17) _a_	6.87 (0.69) _b_	14.04 (1.78) _c_	83.36***
Attitudes towards violence				
Culture of violence	2.05 (0.12) _a_	5.80 (0.54) _b_	13.75 (1.80) _c_	91.07***
Reactive violence	5.27 (0.22) _a_	11.49 (0.65) _b_	16.21 (1.14) _c_	168.13***
Impulsivity traits				
Positive urgency	3.61 (0.14) _a_	6.37 (0.44) _b_	9.82 (0.73) _c_	104.76***
Negative urgency	4.14 (0.17) _a_	7.75 (0.55) _b_	9.69 (0.80) _b_	84.36***
(lack of) premeditation	5.23 (0.16) _a_	7.17 (0.52) _b_	7.76 (1.00) _b_	18.44***
(lack of) perseverance	4.52 (0.16) _a c_	6.47 (0.40) _b_	6.87 (1.24) _b c_	22.69***
Sensation seeking	4.32 (0.17) _a_	6.88 (0.53) _b_	9.38 (0.73) _c_	66.73***
Psychopathic traits				
GM	3.46 (0.19) _a_	7.30 (0.63) _b_	13.71 (1.44) _c_	83.54***
CU	3.19 (0.15) _a_	5.25 (0.64) _b_	11.24 (1.41) _c_	43.31***
IMP	5.81 (0.19) _a_	6.62 (0.62) _b_	13.94 (1.43) _c_	66.42***
Behavioral outcomes				
Rule-breaking behavior	1.00 (0.12) _a_	5.26 (0.65) _b_	11.49 (1.77) _c_	79.90***
Theft	0.38 (0.07) _a_	1.77 (0.41) _b_	8.85 (1.62) _c_	40.61***
Vandalism	0.62 (0.08) _a_	2.59 (0.50) _b_	8.72 (1.66) _c_	40.71***
Drug problems	1.19 (0.13) _a_	3.39 (0.71) _b_	11.71 (2.57) _c_	27.35***

Note. GM = Grandiose/manipulative; CU = Callous/unemotional, IMP = Impulsive/irresponsible. Means with different subscripts (a, b, c) were significantly different (p < .05) in post hoc pairwise comparisons (subscript *a* represents the lowest score/s in the analyzed indicator)

*** p < .001

## Discussion

The current study sought to provide further support for the distinction between RA and PA at a variable-based and person-based level considering two different samples of adolescents. Overall, the results from a variable-based approach suggest that RA and PA are distinct factors which show differential associations with a set of psychosocial correlates and behavioral outcomes. While RA is strongly associated with impulsivity facets, PA is more related to antisocial peers, psychopathic traits, and antisocial behavior. However, the distinction between both functions of aggression from a person-based approach is not so clear. According to the results of the LPA, three different groups of adolescents were identified, classified into low aggression, moderate RA, and mixed aggression. However, differences among subgroups in psychosocial correlates and behavioral outcomes are mainly quantitative, indicating a severe risk profile in those adolescents scoring higher in both RA and PA. Despite some minor differences, the results were replicated in the sample of juvenile offenders and community youth.

### RA and PA Distinction from a Variable-Based Approach

The results of the factor analysis supported the original two-factor structure of the RPQ proposed by Raine et al. [6], both in the forensic and community samples of adolescents. Although good fit indices were found, there was no measurement invariance across samples, indicating that the factor structure, factor loadings, and item intercepts are not held in these two samples of juvenile offenders and community youth. Previous studies replicated the two-factor model of the RPQ in community [6, 24, 33] and detained adolescents [12, 21], and even found support for measurement invariance across samples of detained and community girls [34]. The lack of measurement invariance across samples in the current study indicates slight differences in the performance of the RPQ when using samples of juvenile offenders and community youth.

The analysis of the associations between RA and PA with psychosocial correlates and behavioral outcomes were conducted separately for the two samples, however, the results showed a similar trend in forensic and community youth. At a bivariate level, both functions of aggression were related to most of the correlates, however, when the correlations were controlled for the other function of aggression, some differential associations emerged. In line with expectations, RA remained associated with impulsivity traits and showed strong relationships with the reactive violence facet of attitudes towards violence. On the other hand, PA remained associated with antisocial peers, psychopathic traits, antisocial behavior, and the attitudes towards violence facet of culture of violence. These findings are in accordance with previous studies that showed differential associations with a set of psychosocial correlates [5, 14] and support the hypothesis of the actual distinction of RA and PA from a variable-based approach.

### RA and PA Distinction from a Person-Based Approach

The hypothesis which stated that three groups of adolescents would be identified based on their scores on RA and PA was confirmed. This result was replicated in the sample of juvenile offenders and community youth. The three groups (i.e., low aggression, moderate RA, and mixed aggression) resemble those found in previous studies with adolescent samples, including detained [21] and community youths [17, 18, 20]. In line with prior findings, no “pure proactive” profile emerged, which suggest that PA does not occur without RA and, therefore, must be considered in interaction with RA [9, 15, 20]. Some authors tried to explain this finding suggesting that it is possible that this “pure proactive” group exists in community population but not in clinical or forensic samples [8], however, our results indicate that the same behavioral profiles can be identified in both samples, with some differences in the composition of the groups. Specifically, the low aggression group is larger in the community sample, whereas the moderate RA and mixed aggression groups are larger in the forensic sample, which is not surprising given the juvenile offenders tend to show a more antisocial profile.

The aggressive profiles mainly differ quantitatively in RA and PA, being the mixed aggression group the one scoring higher on both functions of aggression whereas the low aggression group display the lowest scores. Noteworthy, the magnitude of the differences among subgroups was larger for PA, which means that the levels of PA highly differ among subgroups while the three groups score in RA, though to a different extent. As some authors have pointed out, PA must be considered as a marker of more severe aggression rather than a distinct aggressive subgroup [15, 20]. This result has relevant implications for clinical practice, especially for the adaptation of risk assessment instruments and interventions with adolescents in the juvenile justice system [10, 23].

The three aggressive profiles do not differ in terms of gender and age, except for the low aggression group in the community sample that included more females than the moderate RA. These results are in line with previous studies that did not find age or gender differences among subgroups [8, 18], however, they differ from others which found differences in aggressive profiles between boys and girls, with older children being overrepresented in the mixed group [16]. On the other hand, our results support the proposed severity model hypothesis, which stated that individuals in the mixed aggression group are more prone to worse behavioral disruptions and psychosocial correlates [21]. Quantitative differences were found in all the psychosocial correlates and behavioral outcomes, with the mixed aggression group showing a higher risk in all the variables. This result is in line with previous studies and further support the hypothesis of PA as a marker of severity [8, 15]. Notwithstanding, no significant differences were found between the mixed aggression and the moderate RA group in certain impulsivity facets and/or psychopathic traits and drug problems, suggesting that although the mixed aggression is a more disturbed group, the moderate RA must also be considered for intervention.

### Limitations

The results of this study must be interpreted considering some limitations. First, all the variables were measured by means of self-report questionnaires, therefore, results might be partially influenced by shared method variance. Future studies must consider different sources of information as well as different methods to ensure the validity of the results. Second, the questionnaire used to gather data on aggression only assesses functions of aggression, however, and as prior studies have pointed out [9, 16, 20], both forms and functions of aggression may have an influence on the identification of aggressive profiles. Thus, considering not only RA and PA but also their interaction with other forms of aggression, such as physical or relational aggression, may help in defining more precise profiles regarding aggression. Finally, although gender differences in the composition of the groups were analyzed, a deeper analysis on gender differences in RA and PA and aggressive profiles is needed. Previous studies found gender differences both in aggression and its psychosocial correlates [14, 35], which might be partially influencing the results.

## Summary

The distinction between RA and PA has been widely supported from a variable-based approach, however, the identification of distinct aggressive profiles at a person-based level is not well-stablished. In order to replicate previous findings, this study used more robust analytical techniques (i.e., LPA) for the identification of aggressive profiles in two different samples of adolescents (i.e., juvenile offenders and community youth). Differences in a set of psychosocial correlates from different domains (e.g., peers, attitudes, personality) and behavioral outcomes were considered for the analysis of the distinctiveness of RA and PA. The current results provide support for the distinction of both functions of aggression at a variable-based level. Specifically, the original two-factor model of the RPQ was replicated in both samples of juvenile offenders and community youth, and differential associations with psychosocial correlates and behavioral outcomes emerged when controlling for the other function of aggression. However, from a person-centered approach, a “pure proactive” group of aggressive adolescents was not identified but three different aggressive profiles, namely a low aggression, moderate RA, and mixed aggression. The mixed aggression group showed the highest scores in most of the psychosocial correlated and behavioral outcomes, supporting a severity model of aggression. These results were replicated in both samples of juvenile offenders and community youth, suggesting that PA rarely occurs in absence of RA regardless of the antisocial profile. Overall, the current findings indicate that although RA and PA are distinct factors of the same construct, they usually co-occur within individuals. Therefore, PA should be considered as a severity marker rather than an independent subgroup of aggressive adolescents. These results have implications for risk assessment and intervention with juvenile offenders, as well as for prevention of antisocial behavior in high-risk community youth.

## Data Availability

The data are available upon request of the corresponding author.
